# Case of Peculiar Vocal Sound Issuing from a Corpse

**Published:** 1847-05

**Authors:** 


*Case of Peculiar Vocal Sound issuing from a Corpse.*—By E. Crowell, M. D.

The circumstances attending the following case being novel to some of my friends and myself, have induced me to communicate it for insertion in the columns of this Journal. About 2 A. M., February 6th, I was called to the house of Mr. A., corner of 10th Avenue and 19th Street. At about 12 o'clock, Mrs. A. had died; of what disease, I am not informed, as I have not seen the attendant physician, Dr. Dods. I was told by the friends, that she had had uterine hæmorrhage, an inflammation of some of the abdominal viscera. When I arrived at the house, I met four or five females in the hall, who informed me that they were a few minutes previously engaged in laying out the body, when in raising the shoulders a groan was uttered. Very naturally, they dropped it and fled. Under these circumstances I found them, and soon prevailed upon them to re-enter the apartment, where I discovered the body extended upon blankets on the floor. I examined it, and found every characteristic mark of death. I then requested two of the women to raise the shoulders and head, which they did, when a sound escaped from the mouth precisely similar to a short groan. This was repeated a number of times, and invariably with the same effect. I then placed my hands, one on each side, over the lower portions of the ribs, and elevated and depressed alternately the chest as much as possible. This movement reproduced the same sound, only that it was shorter. Pressure upon various parts of the abdomen produced no effect, excepting immediately over the epigastrium, when a similar, but a weaker sound was heard. The body was considerable swollen and distended, and was that of a large woman, probably weighing 170 pounds or more. The cause of the noise, I have no doubt, was a disengagement of air from the stomach, by the pressure made upon the abdomen. But what could have produced such a peculiar vocal sound, I cannot conceive, unless we suppose the muscles of the throat, which are concerned in the formation of the voice, to have retained their tone and elasticity for two hours or more after death. It was a novel case to myself, and perhaps may possess that feature in the consideration of some others.—*N. Y. Annalist.*

*February* 19, 1847.

